# Team social cohesion, professionalism, and patient-centeredness: Gendered care work, with special reference to elderly care – a mixed methods study

**DOI:** 10.1186/s12913-017-2326-9

**Published:** 2017-06-02

**Authors:** Ann Öhman, Britt-Inger Keisu, Birgit Enberg

**Affiliations:** 1 Umeå Centre for Gender Studies, Umeå University, SE-90187 Umeå, Sweden; 2 Public Health and Clinical Medicine; Epidemiology and Global Health, Umeå University, SE-90187 Umeå, Sweden; 3 Sociology, Umeå University, Umeå, Sweden; 4 Community Medicine and Rehabilitation; Physiotherapy, Umeå University, SE-901 87 Umeå, Sweden

**Keywords:** Work satisfaction, Elderly care, Nurses, Occupational therapists, Physiotherapists, Gender, Mixed methods, Clients, Power relations, Sweden

## Abstract

**Background:**

Healthcare organisations are facing large demands in recruiting employees with adequate competency to care for the increasing numbers of elderly. High degrees of turnover and dissatisfaction with working conditions are common. The gendered notion of care work as ‘women’s work’, in combination with low salaries and status, may contribute to negative work experiences. There is abundant information about the negative aspects of elderly care health services, but little is known about positive aspects of this work. The study aim was to investigate work satisfaction from a gender perspective among Swedish registered nurses, physiotherapists, and occupational therapists, focusing specifically on healthcare services for the elderly.

**Methods:**

A mixed methods approach was adopted in which we combined statistics and open-ended responses from a national survey with qualitative research interviews with healthcare professionals in elderly care organisations. The survey was administered to a random sample of 1578 registered nurses, physiotherapists, and occupational therapists. Qualitative interviews with 17 professionals were conducted in six elderly care facilities. Qualitative and quantitative content analyses, chi^2^ and constructivist grounded theory were used to analyse the data.

**Results:**

There was a statistically significant difference in overall work satisfaction between those who worked in elderly care and those who did not (64 and 74,4% respectively, *p* <0.001). Nine themes were derived from open-ended responses in the questionnaire. The qualitative interviews revealed four prominent storylines: ‘Team social cohesion’, ‘Career development and autonomy’, ‘Client-centeredness’, and ‘Invisible and ignored power structures’.

**Conclusions:**

The results show the complexity of elderly care work and describe several aspects that are important for work satisfaction among health professionals. The results reveal that work satisfaction is dependent on social interrelations and cohesion in the work team, in possibilities to use humour and to have fun together, and in the ability to work as professionals to provide client-centered elderly care. Power relations such as gendered hierarchies were less visible or even ignored aspects of work satisfaction. The storylines are clearly linked to the two central discourses of professionalism and gender equality.

## Background

This article focuses on professional discourses of work satisfaction in Swedish healthcare and specifically among registered nurses, occupational therapists, and physiotherapists working in elderly care. The increasing numbers of elderly in the population has put strains on elderly care organisations and led to problems in recruiting sufficient numbers of employees with adequate competence. It has become difficult to offer professional development to existing staff and to retain them in the organisations. Further, the Swedish healthcare workforce is itself ageing, and a shortage of personnel of all kinds has been seen as elderly care seems to have become a less viable arena for career development among health professionals [1]. Substantial international research has shown an array of problems with staff in elderly care, such as high degrees of turnover, problems with retaining high competence, dissatisfaction with working conditions, and limited opportunities to develop professionally [1–3]. The reasons these problems exist are not always described clearly in the literature. However, Hill [4] found clear associations between work satisfaction and intent to stay at the workplace among nurses. In a meta-analysis including 62 studies from 1980 to 2009 of predictors for job satisfaction among frontline registered nurses, Saber [5] concludes that autonomy and job stress were found to be moderate predictors of satisfaction. In a cross-sectional survey of 833 nurses in a university hospital in Sweden, Gardulf et al. [6] found the following factors to be important for work satisfaction: good leadership, the ability to use one’s professional competence, opportunities to develop competence, support for research, professional career development, and continuous dialogue with managers. However, the existing research on work satisfaction among health professionals primarily deals with nurses and nurses aids, whereas fewer studies include other professions such as physiotherapists and occupational therapists, and very few focus specifically on elderly care. In labour market prognostications, Statistics Sweden demonstrated how the demand for registered nurses, occupational therapists and physiotherapists will exceed the supply. This will be especially problematic in elderly care, as the ageing population is growing quickly [7]. With this as background, studying how members from these three professions define and experience work satisfaction, and what would make them stay and develop in elderly care is important.

In previous studies on professional development and work satisfaction among recently graduated registered nurses, physiotherapists, and occupational therapists, elderly care was one of the least preferred areas for a professional career, although many were working in elderly care at the time of the studies [1, 8, 9]. There are similar patterns of dissatisfaction with working in elderly care among the three professions, and statistically significant associations between limited opportunities for professional development and work dissatisfaction. These results indicate that elderly care is often a place where new graduates start their professional careers, but they eventually leave elderly care for work in other areas. These issues prevent elderly care from becoming a sustainable arena for professional development and might even lead to lower quality care. Accordingly, there is a body of knowledge that provides rich information about the negative aspects of health services for the elderly, whereas little is known about positive aspects for work satisfaction among health professionals in elderly care. Thus, it is important to scrutinise how elderly care can become an attractive career choice for health professionals in order to provide high-quality care and rehabilitation to the ageing population. In order to avoid repeating the well-known negative aspects of elderly care work, we have chosen to focus on positive aspects of elderly care work among Swedish registered nurses, physiotherapists, and occupational therapists.

The Swedish labour market is clearly gender segregated. Healthcare organisations are dominated by women employees. Women are found at all levels in the work hierarchy, although more men possess higher level posts. Scholars in gender theory demonstrate how healthcare work is linked to traits associated with women and expected femininity. For example, femininity is associated with a caring disposition, emotions such as empathy, communication and problem-solving abilities, and capacities to understand others and care for their needs [10, 11]. Ideas about gender and work depict two opposites in which women and femininity are often subordinated to men and masculinity [12]. The female gender coding of work is thus interwoven in complex gender power structures that lead to lower pay, less status, and worse working conditions in healthcare than equivalent male-coded positions. In nursing, physiotherapy, and occupational therapy, men are the minority (although more men are entering the physiotherapy profession). Nursing, occupational therapy, and physiotherapy professions are therefore often labelled ‘women professions’.

These professions have historically been subordinated to the male-coded medical profession, in both healthcare organisations and in training programmes [13, 14]. The gendered and symbolic meaning of care work in combination with low salaries and status might be some of the reasons for negative work experiences in elderly care among the three professions. In addition, the university faculty in these training programmes are also predominately women. However, the academisation process of the three professions that started in the 1970s has led to increased professional autonomy and independence from the male-coded medical profession [13]. Students at these university programs are now taught to engage in professional knowledge development through generic academic skills such as critical appraisal of recent research literature, evidence-based practice, and evaluation of their own practice.

Gender and power relations are factors that are often neglected in research on work satisfaction among healthcare professionals, and this is specifically true when it comes to work in elderly care. To our knowledge, no studies exist that focus on work satisfaction and gender in Swedish elderly care. In order to fill this knowledge gap, we have here performed a gender analysis to study positive aspects and shifting narratives of elderly care work that might lead to increased work satisfaction and a more sustainable work organisation with less turnover and improved professional development. Thus, the aim of this study was to investigate aspects on work satisfaction from a gender perspective among Swedish registered nurses, physiotherapist, and occupational therapists, focusing specifically on elderly care.

## Methods

Statistics and open-ended responses from a national survey were combined with qualitative research interviews with healthcare professionals in a mixed methods research design [15, 16]. Mixed methods research is characterized by collection of qualitative and quantitative data in the same study, and integration of these data at some stage of the research process [16]. This study is part of a larger project on work in elderly care that focuses on 1) how elderly care can recruit and retain health professionals, and 2) how elderly care can become an area of sustainable career development. The national survey derives from a cohort study of registered nurses, physiotherapists, and occupational therapists. In the current study, 10-year follow-up data on work satisfaction is used. The research question builds on the previous research from the larger project. Because we found that elderly care was not a preferred area for professional development in the baseline and 10-year follow up (work in progress), we wanted to expand our understanding of work satisfaction in elderly care.

The mixed methods study design allows combination of large scale survey data with qualitative interview data from a small sample. The two data sets were collected sequentially, and the qualitative interview study guide builds on results from the survey. This mixed methods design is used to confirm, complement, and expand information as described in the mixed method research approach of Andrew and Halcomb [16]. This strategy enhances the quantitative questionnaire findings by adding qualitative interview data, and an expansion strategy was used to broaden the study scope. These procedures are all consistent with a mixed methods research approach.

### Sampling, data collection and informants

In accordance with a mixed methods approach, data collection was performed in two stages in which the first step informed the second. The first step consisted of a 2012 national postal survey of three random, stratified samples of the registered nurses, physiotherapists, and occupational therapists who graduated from university in 1999, and thus had been working in their respective professions for approximately 12 years. The sample of 1578 individuals constitutes 48% of the 3300 that graduated in 1999 and includes the total population of physiotherapists and occupational therapists, and one third of the nurses. Baseline and 10-year follow-up data are available for this cohort, but the current study only uses the 10-year follow-up data.

The survey was conducted in collaboration with Statistics Sweden who drew the samples from population registers. The samples were stratified for sex and mirrored the proportion of men and women in each profession. Data were merged into one SPSS file. The questionnaire included questions about socio-demographics, work satisfaction, and career plans. The survey measured aspects of work satisfaction in several ways. We will focus here on the question on overall work satisfaction and the open-ended, free-listing question about work satisfaction. Overall work satisfaction was estimated from the question “Are you satisfied with your work?” on a four-point modified Likert scale ranging from ‘Not at all satisfied’ to “Very satisfied”. To gather more detailed information about aspects that might constitute work satisfaction, we utilized the technique of free listing [17]. The free-listing question was stated as follows: “List three aspects of work that fundamentally contribute to work satisfaction”.

Because we saw clear differences in the questionnaire between reported work satisfaction for those who were working in elderly care and those who were not, we turned to elderly care organisations for further elaboration on work satisfaction. The analysis and the results from the national survey thus constituted a point of departure for the qualitative interviews. We developed a thematised interview guide based on the findings from the survey to use for the qualitative interviews. The interview guide elaborated on the major themes from the free listing such as the importance of the work team, appropriate work tasks, leadership, career development opportunities, rewards, influence, and decision making. We also included questions about gender relations and power hierarchies. Our main aim with the interviews was to deepen the understanding of circumstances that contribute to work satisfaction and positive work experiences in elderly care. The interviews lasted 60–90 min and were voice-recorded and transcribed verbatim. All interviews were conducted by BK, BE, and AÖ, and one researcher on the team took the main responsibility for steering the interview. A constructivist grounded theory approach was used, with an emergent research design as described by Lincoln and Guba [18]. An abductive process was used in which we oscillated between data collection and interpretation. Number of interviewees was not determined beforehand, instead data collection ended when saturation was reached, i.e., no added information was obtained during the last two or three interviews.

Confidentiality in the national survey was guaranteed in that Statistics Sweden conducted the sampling and data collection procedures and provided us with an SPPS file without person numbers or other identifying characteristics. In the interviews with professionals in elderly care, it was important to assure confidentiality so that informants could speak freely about work conditions, management, and the organisation they worked for. Because the interviews were conducted throughout the country, it is not possible to trace specific organisations or individuals in the interview data. However, within the work places it was sometimes difficult not to reveal who from the staff was being interviewed during our visits. Therefore, we do not reveal any details about the specific workplaces, and when citing the informants, we do not indicate their profession. They only have a serial number, which is not linked to their elderly care facility.

We recruited elderly care organisations through suggestions from the respondents in the national survey who were asked to indicate workplaces in elderly care that they considered to be good workplaces. From those suggestions, we visited six elderly care facilities throughout the country in which we interviewed the health professionals. The facilities were chosen so as to cover public as well as private elderly care organisations with varying focus and with geographical diversity that included larger cities as well as smaller municipalities. The elderly care organisations varied in organisational structure and in ownership and included two nursing homes, one rehabilitation centre, and three geriatric hospital wards. One organisation was run by a private company, and five were run as public organisations (three by local municipalities and two by regional county councils). We conducted on-site interviews with a purposive sample of 17 healthcare professionals, including 14 women and 3 men. Five were nurses, six were physiotherapists, and six were occupational therapists (for a more specific description of the sample, see Table 1).

**Table 1:** Characteristics of informants and organisations in the qualitative research interviews

Profession	Sex	Ownership and type of organisation
N	F	Public (Municipality), Nursing home 1
OT	F	Public (Municipality), Rehabilitation centre
PT	F	Public (Municipality), Rehabilitation centre
OT	F	Public (Municipality), Rehabilitation centre
PT	M	Public (Municipality), Rehabilitation centre
OT	F	Public (County council), Geriatric ward 1
PT	F	Public (County council), Geriatric ward 1
PT	F	Public (County council), Geriatric ward 2
OT	F	Public (County council), Geriatric ward 2
N	F	Public (County council), Geriatric ward 2
OT	F	Private company, Geriatric ward 3
PT	F	Private company, Geriatric ward 3
N	M	Private company, Geriatric ward 3
N	F	Private company, Geriatric ward 3
N	F	Public (Municipality), Nursing home 2
OT	F	Public (Municipality), Nursing home 2
PT	M	Public (Municipality), Nursing home 2

*N* Nurse, *OT* Occupational Therapist, *PT* Physiotherapist, *F* Female, *M* Male

### Data analyses

Statistical comparisons from the national survey regarding work satisfaction between those who were working in elderly care and those who were not were performed using chi^2^-tests. The open-ended responses of the free listing were analysed with qualitative and quantitative content analysis [19], which here means that we counted the frequencies of responses, grouped similar responses together, and qualitatively thematised them into themes. It is important to note that the frequencies presented from the free-listing procedure represent a sum of all aspects mentioned that adhere to a certain theme, i.e., they do not represent individuals and they do not distinguish between those working in elderly care and those working elsewhere. The analyses were guided by a gender theoretical approach that takes ‘doing gender’ theory as its point of departure. Gender is defined here as being produced and reproduced in a number of ways in everyday life. This includes working life and work organisation. Healthcare work is considered as changing and changeable, and being socially and culturally constructed, all of which create interwoven and complex processes in the workplace. Ackers work [20, 21] is used as the analytical framework for the study because we agree that gender is embedded in all workplace organisational processes. Gendered processes are concrete activities such as what people say and do, as well as how they think about different activities. The construction of gender has material and ideological implications and constraints at the workplace level. Constraints set work limits in a number of ways and on a daily basis. For example, the construction and reconstruction of gender segregation in healthcare organisational work limit the action of particular employees, both women and men. The production of gender divisions, creation of symbols regarding content of work, interactions between individuals in the workplace, and worker understanding of gendered structures have been described as four sets of analytically distinct, gendered processes in organisations. In the qualitative interview analysis, this framework was used. The focus was on how the informants reasoned about work satisfaction, their work roles, the content of work, and how they describe and express their interactions with colleagues and patients.

A constructivist grounded theory approach was used to analyse the interview texts [22]. This enabled us to examine how the professionals in elderly care constructed meanings related to work satisfaction. Through this approach, we explored and interpreted participants’ meanings and taken-for-granted discourses with the goal to construct an interpretive representation of their social worlds [22]. In accordance with a constructivist grounded theory approach, the process was abductive and oscillated between data collection and analyses in order to use emerging results from previous interviews to inform subsequent interviews [22]. We analysed the interview data in three major steps. First, we conducted a separate open coding of the texts. By constantly comparing the codes, some preliminary storylines started to emerge that consisted of groups of codes that referred to a similar topic. The concept of ‘storylines’, as described by Jones [23], refers here to the ways in which the informants talked about work in elderly care. Second, we performed a selective coding in which we constantly compared the open codes from the separate code lists, meaning that codes were reread and selected to refine the storylines. The whole coding process resulted in four prominent storylines, of which three represent the most central and recurrent meanings of work satisfaction in elderly care among the informants. The fourth storyline mirrors hidden, or unaware, aspects of work in elderly care organisations. In the third and final step, we constructed a conceptual model in which the four storylines are linked to each other. The final result is a negotiated outcome developed jointly among the three researchers. In the discussion, we relate our conceptual model to two major discourses: professionalism and gender equality.

### Methodological considerations

We judge the chosen research design of mixed methods as appropriate because our aim was to broaden and deepen the understanding of work satisfaction among health professionals in elderly care. The confirmation strategy indicates that the qualitative and quantitative datasets focused on the same phenomenon: work satisfaction among healthcare professionals. The complementary strategy was used to complement the national survey with qualitative data. The expansion strategy was used to broaden the understandings of ways in which health professionals’ reason around work and work satisfaction in elderly care. The first dataset (quantitative survey) informed the second (qualitative interview data), and the results revealed similar, but not identical, patterns. The sample of the survey is large, since it constitutes almost 50% of the total population of nurses, physiotherapists and occupational therapists that graduated in 1999. Possible biases were minimized by having the sample drawn from population registers by Statistics Sweden. Statistics Sweden also calculated the sample size, and calculated and randomized the sample, including a procedure for drop-outs. A postal survey response rate of 65% is considered high in Sweden. The analyses were conducted in sequence, and the quantitative dataset was analysed first and findings from that analysis were used to develop the study design for the interviews and the thematic interview guide. Participants in the national survey did not all work in elderly care, and this enabled us to compare reported work satisfaction among those who worked in elderly care and those who did not. Our main aim was not to compare the three professions with each other, but rather to treat them as one group of healthcare professionals with similar educational background, autonomy and competencies. Further, we used a gender theoretical framework to understand these professions as being female-coded professions in a gendered work organisation. We were not primarily interested in comparing women and men, but rather viewing the three professions as groups with gender-coded characteristics that produce certain gendered relations in the work place.

## Results

Most of our findings converged between the questionnaire free-listing and the qualitative interviews, but there were also some divergent results. Although the samples are different, the qualitative results are a confirmation, complement, and expansion of the survey findings. The most important divergences regard the value of, or emphasis on, patient relations. Aspects of, and reflections on, gendered care work and power hierarchies were absent in the free-listing and interviews. The survey results and findings from the qualitative interviews are presented in separate sections.

### The national survey

In total, 1024 health professionals responded the postal questionnaire, yielding a response rate of 65%. The distribution of respondents was 55% nurses, 21% occupational therapists, and 24% physiotherapists. Women made up 88% of the respondents, and 12% were men. In the occupational therapy group, only 2% were men, whereas in the physiotherapist group 23% were men and among the nurses 11% were men. The age distribution for all respondents was 34–65 years with a mean age of 42 years and a median age of 40 years. There was a statistically significant difference in overall work satisfaction between those who worked in elderly care and those who did not (64% of those in elderly care were satisfied, while 74.4% working elsewhere were satisfied, *p* < 0.001). There was no statistically significant difference when comparing work satisfaction between the three professions (*p* = 0.77). Further, we found no significant differences between women and men regarding work satisfaction (0.90). Therefore, during the subsequent qualitative analyses, we did not consider differences between the professions or between women and men.

From the content analyses of the free listing of aspects of work satisfaction, we derived nine themes (Table 2).

**Table 2:** Aspects of importance for work satisfaction among nurses, occupational therapists, and physiotherapists from free-listing in the national survey

No. of responses	Per cent	Themes extracted from content analysis of the responses
720	28.8	Well-functioning work team and collaboration
472	18.9	Stimulating work tasks and independence
471	18.8	Good leadership
329	13.1	Good work environment and a well-organised workplace
212	8.5	Career development possibilities
124	5.0	Economic reward
121	4.8	Influence and insight into the greater healthcare organisation
27	1.1	Humour and joy at work
24	1.0	Good relations with patients/clients
2500	100	

Almost 29% of the free-listing topics highlighted a *Well-functioning work team and collaboration* between colleagues, and this theme included support and respect for each other, being loyal to each other, and helping out when needed. A total of 18.9% of the responses were with regard to the content of one’s work being in line with one’s professional skills and competence. We called this theme *Stimulating work tasks and independence*. As part of this theme, it seems important that jobs offer demanding and qualified work tasks so that the professionals can use their competences. Independence at work is also included in this theme, and independence was reported to be important in order to be able to make well-informed decisions based on one’s professional skills. Responsibility and planning of one’s work were other important aspects of this theme, as well as variation in work tasks. Another fifth of the responses (18.8%) concerned *Good leadership*. This theme dealt with aspects such as having a good manager who provides an open work climate, who supports the employees in their professional ambitions, and who is clear in communication and the information that she/he provides. The manager needs to have good communication skills, needs to be fair, and needs to have the ability to show appreciation. The theme *Good work environment and well-organised workplace* (13.1% of the responses) concerned a sound working environment in terms of physical environment, but especially a well-functioning psycho-social work environment. It was emphasized that less stress and a relaxed work pace were important along with flexible work hours, sufficient staff and resources, and trust in the organisation. Factors concerning *Career development possibilities* constituted another 8.5% of the responses in which opportunities for continuous education and competence development were highlighted. Quite surprisingly, only 5% of the responses concerned *Economic reward*. This was expressed with comments such as “earning a decent salary”, “getting paid for the competence that I have” and “I need to be able to make a living”. Less frequent responses concerned the ability to have *Influence and insight into the greater healthcare organisation* (4.8% of responses), which meant being able to take part in decision-making at higher levels in the hierarchy or at least being able to get information about such decisions. One minor theme concerned the importance of *Humour and joy at the work place* (1.1% of the responses), and only 1% of the responses dealt with patients and clients and expressed that *Good relations with patients/clients* were important for work satisfaction.

### The qualitative interviews in elderly care

From the constructivist grounded theory analyses of aspects of work satisfaction, the following four storylines were extracted: ‘Team social cohesion’, ‘Career development and autonomy’, ‘Client-centeredness’, and ‘Invisible and ignored power structures’. The first three storylines represent meanings of work satisfaction in elderly care among the informants. The fourth storyline – ‘Invisible and ignored power structures’ – mirrors hidden, or unaware, aspects of work in elderly care organisations.

#### Team social cohesion

This storyline mirrors the most central and recurrent meanings of work satisfaction among the informants. It can be seen to cover the most important aspects and views of work in elderly care, and it is constituted by the two main elements of team social cohesion and joy. Parts of this storyline are in line with the responses from the free listing in which aspects of teamwork, collaboration, and career development were emphasised. The storyline deals with an essential feeling of camaraderie as well as the joy of working together professionally. It was argued that social cohesion in the work team is exceptionally important and that it is important for both the internal work climate and for overall work satisfaction. It was further described as important to show solidarity with each other and to be ready to help and support one’s colleagues on the team. Aspects such as respect and an open climate were highlighted. According to this storyline, it seems that social cohesion in the work team, opportunities for collaborative actions and the ability to work in accordance with one’s professional ambition are essential in order to do a good job and to feel safe at work. According to the interviewees, all of this leads to increased trust, which fertilises and supports teamwork.

Another highly valued element in this storyline is the presence of humour and joy in the workplace. Humour and joy only made up 1,1% of the responses from the free listing in the questionnaire, which in turn meant that we did not focus specifically on this in the interviews. However, it was brought up in the interviews as a very important aspect for work satisfaction in elderly care. It was considered critically important to create a work environment where it is accepted to make jokes and use humour. They talked a lot about how they laugh and make jokes while working together. Another aspect of having fun together is to socialise during spare time, which was said to enhance the relations in the work team and to create social cohesion in the team. The interviews showed that shared feelings of joy and humour contribute to building trust in the work organisation and lead to a closer and safer atmosphere in the work team.

Well, we wouldn’t have such good cohesion in the group if we didn’t co-operate. We are *one* group that plays a common role in a way…we really collaborate well and we care for each other! It’s easy to ask questions about anything, even if the question sometimes is a bit stupid. (B4)

Yeah, it’s all about the interplay between us, with those that I work with. It’s important that there is a bit of humour, a bit of joy at work…. and I really think we have that here. You see, we can joke but at the same time be focused on the job tasks. That’s important! …. And then also, I mean, outside work, that we go out and have some fun together, have a meal at a restaurant or whatever, that’s also part of work satisfaction. It becomes much more pleasant if one brings in one’s private life as part of the job, so to speak. And sometimes one can hear somebody singing, and so…I think we have good cohesion here. (D3)

There is an open climate here, for change and for each other. We can discuss things openly, even critical things…how we behave with each other, and when things don’t work. (D2)

#### Career development and autonomy

This storyline deals with issues of professionalism and is analogous to the theme ‘Career development possibilities’ from the free listing in the national survey. The possibility of utilising one’s professional competence was talked about in several ways as a highly valued aspect of work satisfaction. One important factor for work satisfaction is to have opportunities for career development within the workplace. This includes opportunities for continuous education, academic training, and updated knowledge of the latest research on care and rehabilitation for the elderly. Further, professional autonomy was highlighted, and this included the ability to work independently as well as to be able to make informed decisions based on one’s professional skills and competence. In this regard, we found differences between the three professional groups in that the occupational therapists and physiotherapists talked about professional solitude and ambiguity with regard to the healthcare setting or organisation in which they worked. This concept dealt with their professional role and how they experienced being an outsider in relation to the overall elderly care organisation, and it was emphasised that their professional competence is not always utilised or asked for to the degree that they would like. They also regarded this as a disadvantage for the quality of care provided to the elderly patients because they felt that their professional competence was highly needed in such an environment. On the contrary, the nurses seemed to take their professional role for granted and did not problematize any outsider perspective.

Well, you see, work satisfaction is about competence…it’s important to be able to use my professional competence, to use my knowledge and to develop professionally. (E3)

I decide myself on a lot of the content in my work, if I want to see a patient during the morning and perform administrative tasks during the afternoon, or vice versa. That I decide myself, which means that I pretty much direct my work tasks myself. On the detailed level, I mean. So for instance, if I make a home visit to a patient, I can decide together with that patient what he or she will do, I mean, in terms of treatment, exercises, and so…so I control a lot of the work myself. (B6)

I think there are way too few opportunities for competence development. Efforts should be made to change that. I need specific training in order to do a good job here because elderly people and their treatment are different from that of adults or children. But there is no time for updating my knowledge base during work hours, so I have to do that during my spare time, that’s how it is. (F3)

Well, you see, for nurses there are plenty of opportunities for professional development, they can specialise in many areas. But for physiotherapists and occupational therapists, there are no such specialised positions, so if one wants to make a career, well…I guess the only opportunities there are would be to become a manager, or to become a PhD student and do research in academia. (D3)

#### Client-centeredness

This storyline mirrors the ways in which the professionals talked about their major role in elderly care. Good relations with patients were not frequently reported in the free listing in the survey and constituted only 1% of the responses. However, when talking to the informants working in elderly care, it was clearly a very important aspect of work satisfaction. Here, good client relations and patient-centered care were emphasised as major factors for work satisfaction. It was frequently emphasised that their major work task is to focus on the elderly and to work with the elderly and that this job requires something specific because elderly patients are different from other patients due to their frailty and vulnerable life situation. Thus it was argued that the competence for working with the elderly is unique and that it requires a specific competence – a competence that has to be constantly updated in continuous education and career development programmes. A holistic view on elderly people was considered extremely important and something that makes elderly care substantially different in comparison to other types of healthcare organisations. The older person needs to be in focus all the time, several informants claimed, and when working with the elderly there is a need to actually also like old people, otherwise the quality of care will suffer. It is important to be able to increase and support older people’s resources, something that is often challenging, but fruitful even when working with frail people. Rehabilitation was mentioned as a key aspect in elderly care and that there is a need to introduce even more “rehab-thinking” in the work organisation. Positive feedback from the elderly was also mentioned as a strongly satisfying and rewarding part of the work.

I have to say that we are foremost here for the patients. So work satisfaction is not about going around having fun all the time, but indeed, I experience great work satisfaction and joy when I can help and do good for them (the patients). That’s the whole thing with working here, the goal…the encounter with the patient, to solve their problems and see their joy…because it’s from patients that I get positive feedback. Well, I can also get feedback from my colleagues, but it’s still the patients’ satisfaction that makes me most satisfied. Then I can be sure I’ve done a good job, and I get a reward from them. That’s the biggest reward in this job. (B5)

Well, I can say that when working with the elderly, it’s quite often that even a little effort can get a lot back in return. To me, that is positive feedback. (F5)

#### Invisible and ignored power structures

This storyline represents ignorance and avoidance of talking about power structures within the healthcare organisation. Due to our theoretical framework of gender as embedded in all organisational workplace processes, which has implications for power hierarchies in healthcare organisations and healthcare work, and due to the themes from the free listing about control over work and influence on decisions in the larger healthcare organisation, we probed these aspects in the interviews. It was therefore somewhat surprising that when we asked about power relations and hierarchies in elderly care organisation, very little was reflected upon. The most common reflection was that “there are no power hierarchies in this organisation”. It was obvious that they did not really see the power structures, or at least they made them invisible in their talk, or they might have omitted them on purpose. At the same time, there was a lot of talk about dissatisfaction with low salaries and poor income development. The only way to increase one’s salary was to become a manager, some claimed. It was also emphasized that there was lack of transparency in terms of decision making at higher levels in the organisation. In addition, gender aspects of the work were highly invisible and ignored, and when they were reflected upon they mainly dealt with gender equality action plans in the work organisation. However, very little was known about the content of these action plans, and it was stated that these issues are seldom discussed in staff meetings, during coffee breaks, or elsewhere.

We are all the same here! I mean, there is little power and status, I think. I mean, everybody has the same status, so we’re *one* group all together. We may have different backgrounds, but otherwise we’re all the same. We don’t talk that much about these issues, like gender equality, power, or equal treatment, it may have been mentioned sometime, but…I mean, sometimes we may talk about different routines and so….I know there will be a workplace meeting, but no, not so much. Well, we keep saying that we want men to come and work here because we think it becomes…it creates a better work environment. But honestly, I have no idea, but I think that the managers work with this. (B4)

At the moment it’s like this…most of us are women here. But I as a woman I can see that men who work in healthcare are treated equally to women. And I don’t feel any difference and I so much appreciate having male workmates. Men often have different views on things than women have. And I actually think that when there are both sexes at a workplace, the conflicts will be reduced when there are two different viewpoints and when there is communication. (A3)

## Discussion

**Fig. 1:**
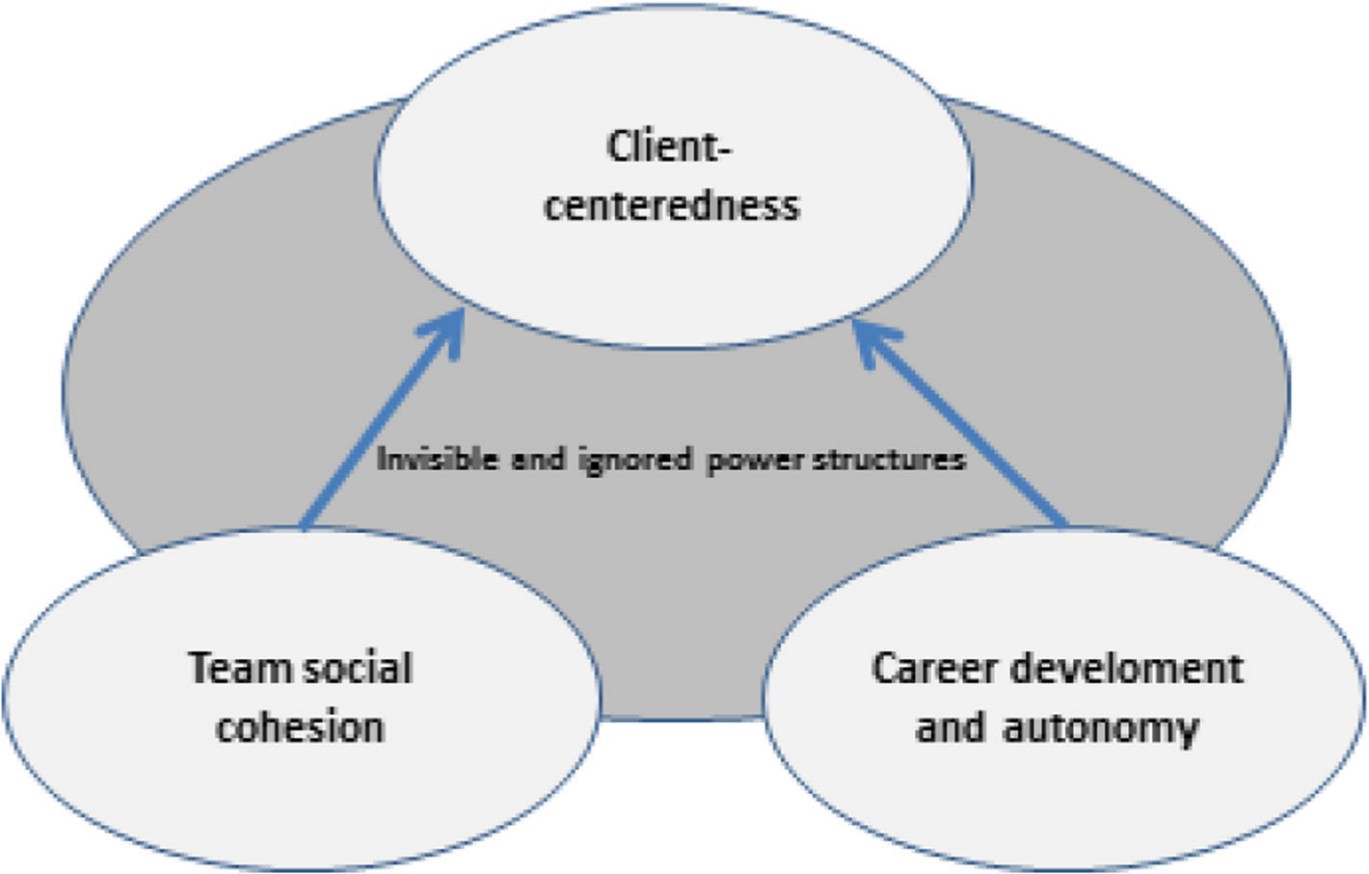
Conceptual model portraying team social cohesion, career development and autonomy as prerequisites for client-centered elderly care

The findings from this study reveal the complexity of elderly care work and describe several aspects that are important for work satisfaction among health professionals. The results from the free listing and the interviews revealed that work satisfaction is dependent on social interrelations and cohesion in the work team, in possibilities to use humour and to have fun together, and in the ability to work as professionals to provide client-centered elderly care. Power relations such as gendered hierarchies were less visible or even ignored aspects of work satisfaction, although they were slightly more pronounced in the free listing than in the interviews. In the final stage of the analyses we constructed a conceptual model that attempts to relate the four storylines to each other (Fig. 1).

The conceptual model depicts how the storylines of ‘Team social cohesion’ and ‘Career development and autonomy’ are closely linked to the storyline of ‘Client-centeredness’. The arrows do not represent causal relations, but should rather be seen as an attempt to visualise that ‘Team social cohesion’ and ‘Career development and autonomy’ form a prerequisite for adopting a client-centered approach to work in elderly care. These three storylines mirror the key elements of work satisfaction among the professionals, whereas the storyline ‘Invisible and ignored power structures’ is placed in the background, implying their lesser importance for work satisfaction as described by the informants. We argue, however, that power relations and hierarchies – whether formal or informal – are present in any given work organisation and that gender and power are important parts of elderly care organisations. We further argue that the storylines are clearly linked to the two central discourses of professionalism and gender equality.

The discourse of professionalism was evident in the ways in which the professionals talked about their work and about what constitutes work satisfaction. This discourse mirrors their professional ambition of having a unique competence that they can use in an autonomous manner in order to make professional decisions. Further, the drive for professional development and a will to constantly update one’s knowledge base is also linked to this discourse. This is very much in line with rather classic descriptions of professions and professionalism. For instance, as early as the 1960s, Parsons [24] and Greenwood [25] described these factors as fundamental features of professions that serve society in an altruistic manner in the interest of all citizens and for the betterment of society. This structural functionalist view of professions was later heavily criticized for not including power struggles, status hierarchies, and gender relations when studying professions [26, 27]. Henriksson et al. [28] demonstrated how professions in the welfare state and in times of new public management are once again treated as manpower, which is a move away from the previously pronounced ‘democratic professionalism’ that had been a marker of the Nordic women-friendly welfare states. They claim that there has been a return to ‘old professionalism’ in which professions such as nursing, physiotherapy, and occupational therapy again become ‘semi-professions’ – as described by Etzione [29] – and subordinated ‘assistants’ in the organisation. The focus on client-centeredness among the informants further mirrors the professional socialisation processes as well as the main obligation of healthcare work, namely that the patient must the placed in the centre of all activities in healthcare work, which in turn implies that the personal interests of individual care workers will be placed in the periphery. This draws on the notion of healthcare work as a calling, something mainly connected to the nursing profession, in which healthcare professionals work for the better of others rather than for their own sake [30]. Bell et al. [31] point out that patient-centeredness is highly connected to the nurse professions and to nurse identity, although the concept is not well-defined in nursing research. We saw however no concrete differences in the ways that the informants from the three professions talked about client-centeredness. It was a common theme throughout the interview material and we interpret this as a gendered notion of healthcare work in which women’s care work for others is put in the centre.

We find it somewhat surprising that power, gender, and inequalities were not on the agenda among the health professionals in this study because gender equality is such an agreed-upon and consensual political discourse in Swedish society. Gender equality is focused on at every level of an organisation, and the Swedish Discrimination Act [32] clearly indicates that workplaces must put effort into working with gender equality and other forms of discrimination and inequalities by formulating actions plans to promote equality. One of the few issues raised by the informants regarding inequalities was the issue of low salaries for health professionals considering their high education. This was viewed as a severe problem in terms of both reward for their hard work and in terms of professional advancement. Therefore, we do not believe that they were completely unaware of gender and hierarchies in healthcare organisations, but it was obviously not a topic that they talked about at work. We interpret this unawareness, or unwillingness to face power and inequalities, as an unconscious strategy to protect oneself in an organisation where there is little possibility to actually influence decisions that are usually taken on higher levels. In gender segregated workplaces, with expected female-coded traits as in the elderly care organisations studied here, it might be too uncomfortable to speak about inequalities and power relations because to do so might disturb the social cohesion in the work team and the “having-fun-together” atmosphere. If issues of power and inequalities are put on the agenda, the social glue between colleagues might disappear, which in turn will jeopardise work satisfaction and the joy of working together in elderly care. Thus, it becomes much more important to focus on one’s inter-personal relations with one’s colleagues and clients. This is in line with Rasmussen [33], who describes how gendered care work has become a greedy organisation in modern societies, in which women care workers struggle to provide good quality care to the elderly at the same time as they are being placed in a squeezed-in position with little power and with no opportunities to influence decisions about resources and time allocation. Rasmussen argues that the gendered expectations of women care workers, as well as the new public management in healthcare organisations with seemingly ‘autonomous’ work teams, make women work harder with little reward in terms of compensation or advancement. We argue, that this relates to professionals in female-coded caring professions, regardless of whether they are women or men.

The importance of the team and of social cohesion in the team was emphasized in the interviews as well as in the free listing. We define this cohesion as the ‘social glue’ between members of the team, a glue that seems to be important in order to be willing to take on new work tasks and to pursue a career within the organisation. Social cohesion has been studied mostly in regard to the welfare state and the larger society, and less in terms of specific workplace conditions. The definitions of cohesion in the literature vary somewhat, but they all deal with individuals’ membership in the group, the duration of this membership, individuals’ attitudes and behaviours, and group performance [34, 35]. Carless & de Paula [35] developed a model for measuring cohesion of work teams in which they include team cohesion, work group characteristics, team effectiveness, job satisfaction, and work group performance. From their model it is obvious that team social cohesion can be linked to work satisfaction. Troth et al [36] related emotional intelligence and communication competence with how students experience social cohesion in group work during their education. In a qualitative interview study of people working in elderly care, Schirmer & Michailakis [37] found something they label ‘lost gemeinschaft’, which they explain as a loss of social cohesion that was prevalent in the past. Their findings stand in contrast to our findings in the current study, because we found that social cohesion was regarded a key factor for work satisfaction in elderly care. Kitson et al [38] found in their review paper of nurses’ communication behaviours that processes and tools that enable social cohesion in work teams have a bearing on performance and client-centeredness.

Another important factor for work satisfaction was the ability to have fun together and to use humour in the work situation. Substantial research has been conducted on joy and humour at work and how this can contribute to a better work environment and how it can affect social relations at work. Zembylas [39] emphasises that historical and ethnographic research has shown “how emotions are experienced through social relations and culture, and thus are managed, not merely expressed, in interpersonal communication in conformity with collective social norms” ([39], p. 444). Holmes [40] states that humour at the workplace has many different meanings, one of which is to build collegiality. In her study of workplaces in New Zealand, Holmes focused on gender constructions and humour at work and showed that humour is important in order to create good relations at work. Creativity, collegiality, and solidarity are all supposed to be fostered when there is the opportunity to use humour in the workplace [41–43]. We also believe that humour and joy can act as protective factors from stress and constraints in the organisation and that these might help the staff to look at their work in a more positive way. Cooper [44] concludes that workplace and inter-personal humour should be viewed as a relational process model constituted by the four distinct processes of effect–reinforcement, similarity–attraction, self-disclosure, and hierarchical salience. She argues that it is important to further investigate how engendered humour is used in the workplace because it might have both negative and positive effects depending on how it is used. Feminist researcher Sara Ahmed [45] describes how emotional norms in a collective either separate us from others or connect us with others. Drawing on Ahmed’s concepts of ‘affective economies’, we label the notions of humour and joy in the interviews as an “economy of joy in healthcare organisations” in which joy and humour are used to provide work satisfaction in an organisation that otherwise offers little in terms of career advancement or economic reward.

## Conclusions

As the ageing population increases in size, and there is a resultant increase in need for high quality care and rehabilitation, the findings from this study are important during recruitment of personnel into elderly care. The three studied professions possess high competency and skills, and they have been trained for autonomous work and decision making. They will therefore be essential in the development of future elderly care. Not to use their competence would be a waste of human capital on organisational and individual levels. The importance of factors such as of a well-functioning work team, stimulating work, independence, and good leadership were consistently discussed as essential to work satisfaction and view of the job. The most obvious difference between the qualitative and quantitative data was the question of patient relations. There was an emphasis on this in the interviews, but it was almost absent in the free listing. Ideas of gendered care work and organisational power hierarchies were surprisingly absent from both data sets. The most important aspect of work satisfaction in elderly care seems to be social cohesion in the work team, which in turn creates trust and collegiality. One important part of this social cohesion is the use of humour and joy during work hours. Another important aspect concerns employees’ opportunities for career development and that the leadership and the organisation support them to work as autonomous professionals. This constitutes the basis for using a client-centered approach with the aged patients, because the elderly need specific care and rehabilitation, including a holistic view of the person in focus. Issues concerning power relations and gendered hierarchies were ignored or made invisible. This leads us to question what would happen if power relations and hierarchies were highlighted and made visible. The tendencies to seek harmony and to avoid conflict are probably rather preservative strategies, which in turn might hamper efforts for change. The health professionals will probably not challenge existing power relations in healthcare organisation but rather reproduce current gender orders and other power hierarchies. This will likely have a long-term negative effect on the ability of elderly care organisations to attract highly competent professionals.
